# Case Report: JAK1 inhibition after dupilumab discontinuation in pediatric atopic dermatitis complicated by pre-existing or emerging vitiligo

**DOI:** 10.3389/fped.2026.1927668

**Published:** 2026-09-22

**Authors:** Lijuan Wang, Zigang Xu, Bin Zhang

**Affiliations:** 1 Department of Dermatology, Beijing Children's Hospital, Capital Medical University, National Center for Children's Health, Beijing, China; 2 Key Laboratory of Major Diseases in Children, Ministry of Education, Beijing, China

**Keywords:** dupilumab, immune-mediated inflammatory disease, JAK inhibitor, pediatric atopic dermatitis, pediatric vitiligo, repigmentation, targeted therapy

## Abstract

**Background:**

Atopic dermatitis (AD) and vitiligo are pediatric immune-mediated inflammatory skin diseases with distinct but potentially overlapping immune mechanisms. Dupilumab targets interleukin (IL)-4 and IL-13 signaling and is effective for moderate-to-severe AD. However, the coexistence, emergence, or progression of vitiligo during dupilumab therapy may create therapeutic challenges when both eczematous inflammation and melanocyte-directed autoimmunity require control.

**Methods:**

We retrospectively reviewed four children aged 6–11 years with AD complicated by pre-existing or emerging vitiligo during dupilumab therapy. Clinical features, timing of vitiligo onset or progression, response to dupilumab, subsequent targeted treatment, follow-up outcomes, and safety data were summarized. Vitiligo was diagnosed clinically and supported by Wood's lamp examination when available.

**Results:**

Two patients had vitiligo before dupilumab initiation, whereas two developed hypopigmented or depigmented lesions during dupilumab treatment. One patient with severe AD showed no obvious improvement after dupilumab; instead, original eczematous lesions became more erythematous and swollen, and new hypopigmented patches appeared around the ankle regions. Dupilumab was discontinued in all patients. Three patients were switched directly to a Janus kinase (JAK) 1 inhibitor. One patient was initially switched to a JAK3/TEC family kinase inhibitor but later changed to a JAK1 inhibitor because of AD recurrence. During follow-up, all patients showed improvement of AD and stabilization or repigmentation of vitiligo. No serious adverse events were observed.

**Conclusion:**

This case series describes a pediatric AD-vitiligo overlap phenotype in which final transition to JAK1 inhibition was associated with improvement of both diseases after dupilumab discontinuation. Causality between dupilumab and vitiligo cannot be established. JAK1 inhibition may be considered in carefully selected children requiring simultaneous control of AD and active vitiligo.

## Introduction

Atopic dermatitis (AD) and vitiligo are common pediatric immune-mediated inflammatory skin diseases (IMIDs). AD is characterized by chronic recurrent eczematous inflammation and pruritus. Type 2 inflammation mediated by interleukin (IL)-4 and IL-13 plays an essential role in AD (1). Dupilumab, an IL-4 receptor alpha antagonist, blocks IL-4/IL-13 signaling and has demonstrated efficacy and safety in children with moderate-to-severe AD (2).

Vitiligo is an acquired depigmented disorder caused by immune-mediated melanocyte destruction in epidermis and/or hair follicle. Its pathogenesis involves melanocyte stress, cytotoxic CD8^+^ T cell, interferon-gamma (IFN-γ), CXCL9/CXCL10-mediated inflammatory recruitment, tissue-resident memory T cells, and JAK/STAT pathway activation (3–6). Although AD and vitiligo are driven by distinct pathogenic mechanisms, their coexistence in children may reflect overlapping immune susceptibility or evolving pediatric IMID phenotypes.

Vitiligo emerging or worsening while taking dupilumab has been reported, but its mechanism isn't still understood (7–11). Possible explanations include an imbalance in T helper pathways and immunomodulation after type 2 blockade, natural progression of melanocyte autoimmunity, or pre-existing vitiligo becoming apparent during follow-up. JAK inhibitors broadly attenuate multiple cytokines implicated in both AD (e.g., IL-4, IL-13, IL-31, and TSLP) (12–14) and vitiligo (e.g., IFN-*γ* and IL-15) (14–16), providing a plausible basis for dual-disease control. Here, we report four children aged 6–11 years with AD complicated by pre-existing or dupilumab- associated vitiligo that were ultimately managed by switching to Janus kinase (JAK) inhibitors.

## Case description

This case series included four children aged 6–11 years diagnosed with AD and vitiligo at Beijing Children's hospital, Capital Medical University from March 2023 to November 2025. AD was diagnosed clinically according to the Chinese pediatric diagnostic criteria for AD proposed by Yao's group, based on pruritus, typical morphology and distribution or atypical morphology and distribution with xerosis, and a chronic or chronically relapsing disease course (17). Disease severity was assessed using eczema area and severity index (EASI), body surface area (BSA), investigator's global assessment (IGA), pruritus numeric rating scale (NRS), and treatment history. EASI ranges from 0 to 72, with higher scores indicating more severe disease; IGA ranges from 0 to 4; and pruritus NRS ranges from 0 to 10, with higher scores indicating more severe itch. All patients had been treated with systemic targeted therapy due to moderate-to-severe AD. Vitiligo was diagnosed based on acquired, well-demarcated hypopigmented or depigmented macules and patches, supported with Wood's lamp examination. Disease activity was evaluated using vitiligo disease activity (VIDA), trichrome lesions, confetti-like lesions, Koebner phenomenon, inflammatory borders (18). Differential diagnoses included post-inflammatory hypopigmentation, pityriasis alba, nevus depigmentosus, tinea versicolor, tuberous sclerosis complex, and other pediatric hypopigmented diseases.

For each patient, de-identified demographic information, AD duration, vitiligo history, lesion distribution, previous treatments, family history of atopy or autoimmune disease, major symptoms, and caregiver concerns were reviewed. In this case series, two patients had vitiligo before dupilumab initiation, whereas the remaining two patients developed vitiligo during treatment with dupilumab. Dupilumab was discontinued in all patients after vitiligo progression or onset. Before and during JAK inhibitor treatment, safety assessments included complete blood count, liver and renal function, lipid profile, infection screening, coagulation function, infection screening, and clinical surveillance for infections. Three patients were directly switched to a JAK1 inhibitor, while one patient was initially switched to a JAK3/TEC family kinase inhibitor and later changed to a JAK1 inhibitor due to recurrence of AD lesions. Concomitant therapies included topical corticosteroids, topical calcineurin inhibitors, emollients, and phototherapy. All systemic targeted therapies were prescribed after discussion with guardians regarding potential benefits, off-label use, safety uncertainties, and monitoring requirements. The clinical characteristics and outcomes of patients are summarized in Table 1.

**Table 1:** Clinical characteristics and outcomes of children with AD-vitiligo overlap.

Variable	Case 1	Case 2	Case 3	Case 4
Age/sex	M	F	M	M
Age at AD onset	1 year-old	6-month-old	3-month-old	2-month-old
Baseline AD severity	EASI 18.8, IGA 3, BSA 8.5%, NRS 8	EASI 12.8, IGA 3, BSA 6.5%, NRS 8	EASI 16.4, IGA 3, BSA 8%, NRS 7	EASI 28, IGA 4, BSA 59%, NRS 9
Vitiligo status before dupilumab	Pre-existing	Pre-existing	No known vitiligo	No known vitiligo
Vitiligo distribution	Left chest and back	Lower abdomen	Left face and neck	Ankle region
Baseline vitiligo activity	VIDA 4, VASI 1.8	VIDA 4, VASI 2.3	Not applicable	Not applicable
Dupilumab regimen	300 mg every 2 weeks, with a double loading dose of 600mg	200 mg every 2 weeks, with a double loading dose of 400 mg	300 mg every 4 weeks, with a double loading dose of 600 mg	200 mg every 2 weeks, with a double loading dose of 400mg
AD response to dupilumab	Improved	Improved	Improved	No obvious improvement: original AD lesions became more erythematous and swollen
Time to vitiligo onset/progression	7 months after treatment with dupilumab	4 months after treatment with dupilumab	6 months after treatment with dupilumab	Hypopigmented ankle lesions developed after 8 weeks with dupilumab therapy
Post-dupilumab targeted therapy	JAK1 inhibitor	JAK1 inhibitor	JAK3/TEC inhibitor, switch to JAK1 inhibitor	JAK1 inhibitor
Concomitant therapy during dupilumab phase	Topical tacrolimus 0.03% and 308 nm excimer for vitiligo; Mometasone furoate and tacrolimus 0.03% for AD lesions	Topical tacrolimus 0.03% and NB-UVB for vitiligo; Topical corticosteroid for AD lesions	Topical tacrolimus 0.03% and NB-UVB for	Topical corticosteroid
Concomitant therapy during JAK inhibitor phase	Topical tacrolimus 0.03% and 308nm excimer for vitiligo; Mometasone furoate and tacrolimus 0.03% for AD lesions	Topical tacrolimus 0.03% and NB-UVB for vitiligo; Tacrolimus 0.03% for AD lesions	Topical tacrolimus 0.03% for hypopigmented and eczematous lesions	Topical corticosteroid for hypopigmented and eczematous lesions
AD outcome	Controlled	Controlled	Controlled after JAK1 switch	Improved after JAK1 inhibition
Vitiligo outcome	Stabilization and repigmentation, VIDA = 1, VASI = 0.045	Stabilization and repigmentation, VIDA = 3, VASI 0.93	Stabilization/repigmentation, VIDA = 2, VASI = 0.39	Stabilization and repigmentation, VIDA 2, VASI 0.23
Follow-up duration	24 weeks	16 weeks	12 weeks	16 weeks
Adverse events and laboratory monitoring during JAK inhibitor therapy	None	None	None	None

AD, atopic dermatitis; F, female; M, male; NB-UVB, narrow-band ultraviolet B.

### Case 1

An 11-year-old boy was diagnosed with AD since the age of 1, presenting with recurrent eczematous lesions, pruritus, and sleep disturbance. Before dupilumab, he had erythematous papules and plaques involving neck, trunk and extremities, with EASI 18.8, BSA 8.5%, IGA 3, pruritus NRS 8, and inadequate response to topical steroids and topical calcineurin inhibitor. He also had 4-year history of vitiligo involving his left chest and back, with baseline activity assessed by vitiligo disease activity (VIDA) −1, vitiligo area score index (VASI) 0.74, and Wood's lamp. No family history of vitiligo and autoimmune disease was reported.

He achieved a marked remission with dupilumab 300 mg every 2 weeks, with a double loading dose of 600 mg. After 7 months of dupilumab exposure, vitiligo progressed with enlargement and new lesions (VIDA 4 and VASI 1.8, Figure 1A). Dupilumab was discontinued after discussion with the guardians and switched to oral abrocitinib (a JAK1 inhibitor) 100 mg daily in combination with narrow-band ultraviolet (NB-UVB) phototherapy twice weekly for vitiligo lesions. After 24 weeks, AD remained controlled, the hypopigmented patches progression stopped, and repigmentation was also observed (VIDA=1, VASI=0.045, Figure 1B). Laboratory monitoring showed normal results, and no serious adverse events (AEs) occurred.

**Figure 1:**
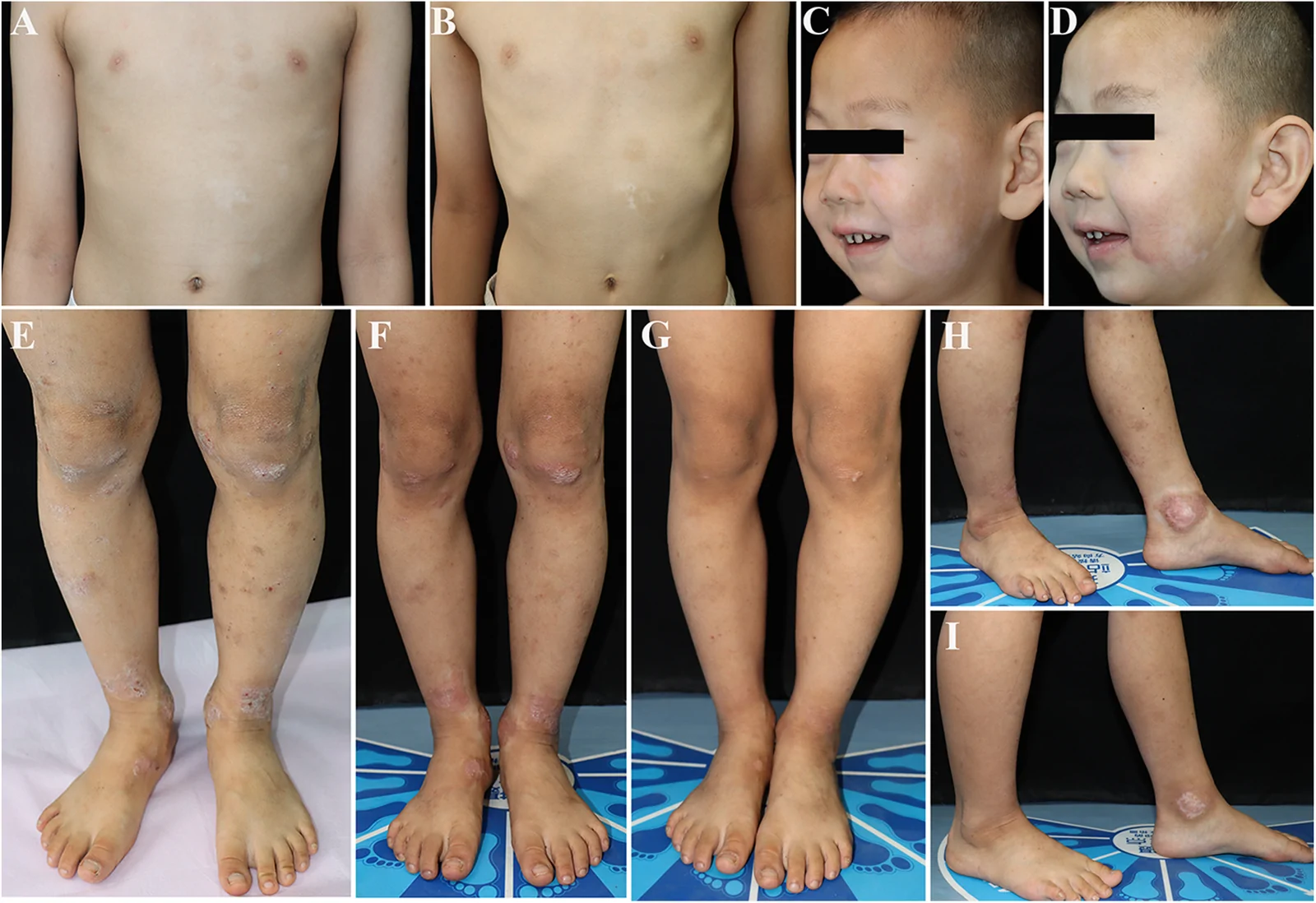
Representative clinical photographs before and after JAK-pathway treatment. Case 1: **(A)** Vitiligo lesions on the left abdomen and back before JAK1 inhibition; **(B)** Repigmentation after treatment with abrocitinib for 24 weeks; Case 3: **(C)** Vitiligo lesions on the left face; **(D)** Atopic dermatitis lesions receded partially, and vitiligo lesions showed repigmentation after treatment with ritlecitinib for 16 weeks. Case 4: **(E)** Baseline clinical photograph before dupilumab initiation showing severe eczematous lesions with erythema and excoriations. **(F)** Erythematous and swollen eczematous lesions during dupilumab therapy. **(H)** Hypopigmented patches around the ankles. Improvement of AD lesions **(G)** and repigmentation **(I)** after 16 weeks of JAK1 inhibition.

### Case 2

A 7-year-old girl was diagnosed with recurrent AD since 6-month-old, presenting with recurrent itchy eczematous lesions. Baseline severity was EASI score of 12.8, IGA score of 3, BSA of 6.5%, and pruritus NRS score of 8, and previous therapies provided insufficient improvement. The patient had a-2-year history of vitiligo involving the trunk, which had remained stable with treatment of tacrolimus and narrow-band ultraviolet B (NB-UVB) phototherapy once weekly for the past year. No family history of vitiligo and autoimmune disease was reported. She was treated with dupilumab, with an initial loading dose of 400 mg followed by 200 mg every 2 weeks.

After 4 months of dupilumab treatment, the patient's eczematous lesions and pruritus markedly improved. However, the depigmented patch on the lower abdomen enlarged, with the appearance of a trichrome pattern, with a VIDA score of 4 and a VASI score of 2.3. Dupilumab was subsequently discontinued, and treatment was switched to upadacitinib accompanied with 0.03% tacrolimus and NB-UVB for vitiligo. After 16 weeks of upadacitinib treatment, the vitiligo lesions remained stable and showed repigmentation, with the VASI score decreasing to 0.93. The AD lesions were well controlled, with EASI score of 0.4, IGA score of 1, BSA of 1.25%, and pruritus NRS score of 0. Laboratory monitoring showed normal results, and no treatment-limiting AEs was recorded.

### Case 3

Case 3 was a 6-year-old boy with AD since 3 months of age, presenting with recurrent itchy eczematous lesions. At the initial visit, physical examination showed erythematous papules and plaques on the trunk and extremities, prodominantly in the flexural areas, with overlying scales, excoriated crusts, mild localized thickening, and lichenification. He was diagnosed with moderate AD, with an EASI score of 16.4, BSA involvement of 8%, an IGA score of 3, and a pruritus NRS score of 7, and previous treatment were inadequate. No family history of vitiligo and autoimmune disease was reported. Dupilumab was initiated at 300 mg every 4 weeks, with a double loading dose of 600 mg. The patient had no known vitiligo before dupilumab. After six months of treatment, new asymmetrical, depigmented, irregular lesions developed on his left face and neck and were confirmed as vitiligo by clinical features and Wood's lamp examination (VIDA=4, VASI=1.06, Figure 1H). Then dupilumab was ceased and switched with oral ritlecitinib (a JAK3/TEC inhibitor) 50 mg daily plus topical tacrolimus 0.03% twice daily. During this treatment, vitiligo stabilized and significant repigmentation after 16 weeks (VIDA=2, VASI=0.39, Figure 1I). However, AD lesions recurred, with worsening eczema involved the face, neck, and flexures, and pruritus. So ritlecitinib was discontinued and replaced by upadacitinib at 15 mg daily. At 12 weeks after switching to upadacitinib, AD improved, vitiligo progression remained suppressed, and repigmentation was observed. Laboratory monitoring showed normal results, and no serious AEs occurred.

### Case 4

An 8-year-old boy was diagnosed with severe AD involving trunk and extremities, which severely affected his quality of life. Physical examination showed erythematous eczematous plaques with excoriations on the trunk and extremities. Disease severity was assessed as severe according to an EASI score of 28, BSA involvement of 59%, an IGA score of 4, and a pruritus NRS score of 9 (Figure 1E). His mother has a history of urticaria. No family history of vitiligo and autoimmune disease was reported. Dupilumab was initiated at 200 mg every 2 weeks, with a double loading dose of 400 mg. Notably, preexisting AD lesions became more inflamed, with prominent erythema and swelling after 8 weeks (Figure 1F). In the meanwhile, newly developed hypopigmented patches appeared around both ankles and gradually enlarged beyond the areas previously affected by eczema (Figure 1H). Therefore, a diagnosis of vitiligo was made with a VIDA score of 4 and VASI score of 0.56.

Given inadequate AD control, inflammatory worsening, and new vitiligo-like lesions, dupilumab was discontinued. The patient was switched to upadacitinib at a dose of 15 mg daily with topical corticosteroid for hypopigmented patches. After 16 weeks of treatment, the eczematous lesions and pruritus were markedly improved, with an EASI score of 2.1, IGA score of 1, and pruritus NRS score of 0 (Figure 1G). Approximately half of the hypopigmented patches on the ankles showed repigmentation, with a VIDA score of 2 and VASI score of 0.23 (Figure 1I). Laboratory monitoring showed normal results, and no serious AEs occurred.

## Therapeutic intervention

Dupilumab was discontinued in all patients after vitiligo onset or progression. Subsequent treatment was individualized according to AD severity, vitiligo activity, previous treatment response, lesion distribution, family preference, and the need to control both diseases. Three patients were switched directly to JAK1 inhibitors, whereas one patient was initially treated with a JAK3/TEC family kinase inhibitor and later switched to a JAK1 inhibitor because of recurrent AD lesions. Concomitant topical therapy and/or phototherapy were used when clinically appropriate. The main therapeutic goals were control of AD, stabilization of vitiligo activity, and repigmentation.

## Follow-up and outcomes

After final switch to JAK1 inhibition, all four patients showed improvement in eczematous lesions and pruritus, as reflected by reductions in EASI, IGA, BSA, and pruritus NRS scores. Vitiligo stabilization and repigmentation were observed during follow-up. Treatment response was assessed by clinical examination and VASI scores, and safety was evaluated through regular laboratory monitoring, including complete blood count, liver and renal function, lipid profile, and infection surveillance. No serious AEs were observed, and no patient discontinued JAK1 inhibition because of treatment-related safety concerns.

## Discussion

This case series describes children with AD complicated by either pre-existing or vitiligo emerging during dupilumab treatment, all of whom were ultimately treated with JAK inhibition after dupilumab discontinuation. All patients achieved clinical improvement of AD and stabilization and repigmentation of vitiligo during follow-up. These observations may be clinically relevant because they illustrate the complexity of managing overlapping pediatric IMIDs, but they should be interpreted cautiously because of the small sample size and observational nature of the report.

AD and vitiligo are generally associated with different dominant immune pathways. AD is commonly linked to type 2 inflammation, IL-4/IL-13 signaling, barrier dysfunction, whereas vitiligo is characterized by melanocyte-specific CD8+ T cells, IFN-*γ*/CXCL9/CXCL10 signaling, and JAK/STAT pathway activation (1, 3–6). Nevertheless, these pathways are not mutually exclusive in individual patients. Epidemiological studies have shown that vitiligo, particularly early-onset vitiligo, is associated with an increased risk of AD, while patients with AD may also have immune-mediated dermatologic comorbidities beyond the canonical type 2 inflammation, including vitiligo and alopecia areata (19–21).

Several previous reports have described new-onset or worsening vitiligo during dupilumab therapy (7–11). Picone et al. reported vitiligo developing during dupilumab treatment in an adult patient with AD, with remission after topical corticosteroids and NB-UVB phototherapy while dupilumab was continued (7). Ren et al. described seven patients with new-onset or worsening vitiligo after dupilumab initiation, with a mean interval of 3.4 months; most patients achieved stabilization or repigmentation with topical therapies and/or phototherapy, and dupilumab was continued in most cases (8). Farinha et al. reported one patient with acral and perimalleolar lesions in whom vitiligo improved after switching from dupilumab to baricitinib, whereas AD subsequently flared and dupilumab was restarted (9). These data support clinical monitoring for pigmentary changes during dupilumab treatment.

The relationship between dupilumab therapy and vitiligo in our patients remains uncertain. In some patients, improvement of eczematous inflammation may make previously subtle pigmentary changes more visible. In others, vitiligo may progress independently according to its natural course. It is also possible that selective type 2 cytokine blockade may alter the relative balance of inflammatory pathways in predisposed patients, potentially permitting relatively increased Th1/Th17 or IFN-*γ*-related activity, but this remains speculative and requires further study (7–11).

Notably, the patient in case 3 had severe AD with poor response to dupilumab and developed worsening erythema and swelling of the original eczematous lesions, together with new hypopigmented patches around the ankles. This course was not consistent with simple unmasking of pigmentary lesions after eczema improvement. Possible explanations include uncontrolled AD, a mixed inflammatory phenotype, or a dupilumab-associated paradoxical eruption, such as psoriasiform or rosacea-like dermatitis (22–24), although the mechanism cannot be determined from clinical observation alone.

The clinical improvement observed after JAK1 inhibition may have several possible explanations. JAK1 participates in cytokine pathways relevant to AD, and IFN-*γ*/JAK/STAT signaling is involved in vitiligo activity (12–14). Thus, JAK1 inhibition is mechanistically plausible in treatment for both AD and vitiligo. In our cases, final treatment with JAK1 inhibitors was followed by improvement of eczema, reduction of pruritus, stabilization and repigmentation of vitiligo. However, these findings should be further studied, as the contribution of concomitant topical therapy and phototherapy cannot be excluded. Topical corticosteroids, topical calcineurin inhibitors, NB-UVB, and 308 nm excimer light are established treatments for AD and vitiligo (2, 12, 18, 25, 26). Pediatric vitiligo studies suggest that 308 nm excimer light combined with topical tacrolimus may enhance repigmentation, and topical calcineurin inhibitors have also shown therapeutic benefit in vitiligo (27, 28). Therefore, the improvement in our patients cannot be attributed solely to JAK1 inhibition. Instead, JAK1 inhibition may have contributed to simultaneous control of AD and vitiligo as part of an individualized combination treatment strategy. We therefore reported concomitant treatments separately for dupilumab phase and the JAK inhibitor phase in Table 1.

Case 4 provides an additional clinical observation. Ritlecitinib was selected because the immediate priority at switching was rapidly worsening vitiligo. He was observed with marked vitiligo improvement with AD lesions later recurred after 16 weeks. Given its JAK3/TEC selectivity, ritlecitinib may more effectively suppressed IL-15-linked vitiligo activity while providing less complete control of JAK1-dependent AD pathways (e.g., IL-13, IL-31, TSLP) (14). However, no firm conclusion can be drawn from a single case.

These cases have practical implications for clinical monitoring. In children treated with dupilumab, especially those with pre-existing vitiligo or unexplained hypopigmented macules, baseline skin examination and follow-up assessment of pigmentary changes may be useful and essential. New hypopigmented patches should be carefully differentiated from post-inflammatory hypopigmentation, pityriasis alba, and other pediatric hypopigmented disorders. Decisions regarding subsequent application of dupilumab should be individualized according to many factors, including AD control, vitiligo activity, lesion extent, psychosocial burden, available alternatives, and caregiver preference. During systemic JAK inhibitor therapy, regular laboratory monitoring should be conducted.

This report has several limitations, including the small sample size, retrospective uncontrolled design, and heterogeneous clinical courses. Follow-up duration varied across cases, raning from 12 to 24 weeks. This variability may have influenced the apparent degree of repigmentation and AD control, because vitiligo repigmentation often requires longer observation than improvement of eczema or pruritus. Therefore, treatment responses across cases should not be directly compared. Causality between dupilumab and vitiligo onset or progression cannot be established. The contribution of concomitant topical therapy and phototherapy cannot be excluded. Therefore, the observed improvement cannot be attributed solely to JAK1 inhibition, although the temporal association after treatment switching is clinically notable.

In summary, this case series describes a pediatric AD-vitiligo overlap phenotype in which final transition to JAK1 inhibition was followed by improvement of both diseases. Compared with previous reports, our cases are notable for the young age of the patients, the inclusion of new-onset or worsening vitiligo during dupilumab therapy, and the ultimate use of JAK1 inhibition to manage both AD and vitiligo. The findings should be considered preliminary. Larger prospective pediatric cohorts with standardized severity scores, long-term safety monitoring, and translational biomarkers are needed to clarify risk factors, mechanisms, and optimal treatment sequencing.

## Conclusion

This case series highlights an overlapping pediatric immune-mediated inflammatory phenotype involving AD and vitiligo. Vitiligo may pre-exist before dupilumab or emerge during treatment, and the relationship and mechanism remain uncertain. In our patients, dupilumab discontinuation and final switch to JAK1 inhibitors were associated with improvement of both AD and vitiligo. JAK1 inhibition may be considered in carefully selected children with AD-vitiligo overlap who require dual-disease control, with appropriate informed consent, safety monitoring, and individualized risk-benefit assessment.

## Patient perspective

The guardians reported that the coexistence of eczema and white patches caused considerable anxiety because the children experienced both itching and visible skin color changes. In Case 4, the family was particularly concerned because the eczema did not improve after dupilumab and the original lesions appeared more red and swollen, while new hypopigmented patches developed around the ankle. After switching therapy, the families noticed improvement in itching, sleep, eczema lesions, and gradual repigmentation of white patches. They expressed relief that both diseases could be controlled with one treatment strategy, while also understanding the need for regular laboratory monitoring and follow-up.

## Data Availability

The datasets presented in this article are not readily available because of ethical and privacy restrictions. Requests to access the datasets should be directed to the corresponding authors.
